# Antipsychotic off-label use in the 21st century: An enduring public health concern

**DOI:** 10.1080/19585969.2025.2449833

**Published:** 2025-01-10

**Authors:** Hélène Verdoux

**Affiliations:** University Bordeaux, Inserm, Bordeaux Population Health Research Center, Team Pharmacoepidemiology, UMR 1219, Bordeaux, France

**Keywords:** Antipsychotic, off-label use, metabolic, marketing

## Abstract

Soon after the introduction of second-generation antipsychotics, antipsychotic off-label use (OLU) progressively became a common prescribing practice. This evolving practice should be regularly monitored considering the growing number of persons exposed to the adverse effects of antipsychotics. The aim of the present review was to synthesise the literature published over the last 15 years on antipsychotic OLU for mental health symptoms. Observational studies confirm the persisting high rate of antipsychotic OLU prescription in two out of three youths and 30–60% of adults using antipsychotics. Increasing rates of low-dose quetiapine prescriptions for anxiety or sleep symptoms are paradigmatic of the current public health concern regarding antipsychotic OLU. Such prescriptions receive impetus from industry-funded marketing strategies and prescribers’ feeling of innocuousness, with a resulting underestimation of the risk of adverse drug reactions (ADR). However, antipsychotic OLU should be neither trivialised nor demonised since it may be the only therapeutic option in persons with resistant psychiatric disorders or serious ADR with labelled drugs. To reduce the populational impact of antipsychotic OLU, it is necessary to better control the influence of the pharmaceutical industry regarding newly marketed drugs and to better inform prescribers and users about the risks associated with OLU prescribing.

## Introduction

Since the introduction of second-generation antipsychotics (SGA), the growing proportion of persons exposed to antipsychotics has become an acknowledged public health concern (Verdoux et al. 2010; Hálfdánarson et al. 2017). This is mostly due to increased rates of prescribing antipsychotic off-label use (OLU) in youths (Patten et al. 2012; Olfson et al. 2015; Verdoux et al. 2015; Radojčić et al. 2023) and in adults (McKean and Monasterio 2012; Carton et al. 2015; Højlund et al. 2019). A major issue raised by this prescribing practice is the exposure of persons with unapproved indications to the risks of adverse outcomes (Galling et al. 2016; De Hert and Detraux 2018; Verdoux 2018; Ray et al. 2019; Højlund et al. 2021b; Hoekstra and Dietrich 2022; Stogios et al. 2022; Højlund et al. 2023).

OLU encompasses a wide range of situations with respect to OLU criteria (age, diagnosis or contraindications) as well as to the level of evidence concerning the prescribing practices of old drugs, which are documented by a large body of literature, and the OLU of new drugs, which is documented by only a few small open studies (Martin-Latry et al. 2007; Schröder et al. 2017; Wang et al. 2021; Grabitz et al. 2024). Furthermore, the licenced indications of drugs are rather dependent not only on their pharmacological properties but also on the commercial strategies of pharmaceutical companies. On the one hand, the costs of pre-marketing trials offer little incentive to apply for new marketing authorisations for old drugs, as explained by many between-country variations of approved indications (Zhu et al. 2018). On the other hand, promoting the OLU of newly marketed drugs obviously contributes to enlarging the size of the treated population (Tanne 2010; Roehr 2012; Tanne 2012; Wang et al. 2016; Wang et al. 2017).

Antipsychotic OLU is an enduring but evolving public health problem (Verdoux et al. 2010; Hálfdánarson et al. 2017). The literature on this issue should be updated regularly to identify signals requiring increased awareness. The aim of the present review was to synthesise the recent literature on antipsychotic OLU for mental health symptoms.

## Materials and methods

A MEDLINE and PsycINFO search was performed from 2010 through June 2024 using the algorithm ‘antipsychotic AND (off-label OR unapproved OR unlicensed)’ to identify articles published in English or French in peer-reviewed journals. Titles and abstracts of retrieved citations were screened and selected full-text articles were assessed for eligibility by the author of the review. Related references of selected papers as well as systematic reviews and meta-analyses were also examined by the author of the review.

First, to explore the frequency and characteristics of antipsychotic OLU at the level of the population actually treated, studies meeting the following inclusion criteria were included: (i) population-based or multicentre studies exploring OLU in real-life prescribing conditions, (ii) exploring antipsychotic OLU targeting symptoms related to mental health conditions, (iii) reporting estimates of antipsychotic OLU (incidence or prevalence rates, prescription data, etc.). Exclusion criteria concerned: (i) studies conducted in mental health structures for specific populations (Veterans Affairs facilities, etc.), (ii) studies on OLU for conditions approved for at least one antipsychotic drug (schizophrenia, bipolar disorder, etc.); (iii) studies on neurodegenerative disorders or non-psychiatric diseases (cancer, etc.) since distinct reviews would be required to synthetise the large body of literature addressing these issues.

Regarding other issues related to antipsychotic OLU (efficacy, adverse drug reactions (ADR), marketing and regulatory issues, etc.), prior systematic reviews and meta-analyses were given precedence over other articles. Since it was beyond the scope of the present review to synthesise the findings of all studies assessing the effects of antipsychotics in non-labelled indications, the search was focused on OLU for conditions not approved for at least one antipsychotic drug.

## Results

### Literature search

Ultimately, 51 articles published from 2010 to 2024 were included (Supplementary Figure 1) addressing the following issues: (i) frequency of OLU in child and adolescent populations (*n* = 12) (Table 1) or in adult/all-age populations (*n* = 15) (Table 2), (ii) antipsychotic OLU in specific mental health conditions (*n* = 7) (anxiety, personality or sleep disorder); (iii) ADR associated with antipsychotic OLU (*n* = 9), (iv) articles on prescribers (*n* = 4; (v) marketing and regulatory issues (*n* = 8) (total number >51 as some articles are quoted in several sections).

**Table 1.** Studies exploring antipsychotic off-label use (OLU) in children and adolescents.

Authors, year Country	Population	Period	Diagnosis off-label use criteria	Main findings
Pathak et al. (2010) USA	Arkansas Medicaid population Youths <18 yrs with new SGA^1^ use (n = 11,700)	2000–2006	ICD-9^2^ Evidence-based OLU^3^ (RCT^4^, other trials)	OLU without strong evidence: 62% OLU without evidence: 41.3% in all diagnoses (ADHD^5^: 58%; oppositional defiant disorder: 28%; depressive disorder: 28%)
Ronsley et al. (2013) Canada	British Columbia prescription database Youths ≤ 18 yrs with AP^6^ use (2010/11 n = 5791)	1996–2011	ICD-9 No OLU criteria	Most frequent disorders: depressive disorders (12.8%), ADHD (11.7%), neurotic disorders (11.1%)
Burcu et al. (2014) USA	Maryland Medicaid population Youths 2–17 yrs with SGA use (n = 8696)	2006	ICD-9 No OLU criteria	ADHD/ DBD^7^: 49.5% Disorders other than psychosis, ASD^8^ and bipolar: 31.8%
Sohn et al. (2016) USA	National Ambulatory Medical Care Survey and National Hospital Ambulatory Medical Care Survey Youths 4–18 yrs with outpatient visits with SGA prescription	1993–2010	ICD-9 FDA^9^-approved indication	OLU visits: 65% Most frequent disorders: ADHD (24%), psychoses (14%), ‘disturbances’ and neurotic disorders (5%) No psychiatric diagnosis: 15%
Schröder et al. (2017) Germany	German statutory health insurance providers Outpatient youths <18 yrs with AP use (2011 n= 5459)	2004–2011	ICD-10 No OLU criteria	All OLU: 61.0% (2004) to 69.5% (2009); by indication: 42.8% to 66.5%; by age: 11.5% to 13.5% Most frequent disorders: ADHD (52.5%); ASD (19.2%); tic disorder (15.7%)
Candon et al. (2021) USA	Pennsylvania Medicaid-population Youths <22 yrs with AP use (2014 n = 7555, 2018 n = 3959)	2014–2018	ICD-9/10 Approved indications: ASD, BP^10^, psychosis	Slight increase in OLU: 44.4% (2014) to 47.3% (2018) Most frequent disorders: ADHD, depression, adjustment disorders, conduct disorders
dosReis et al. (2022) USA	10% random sample of IQVIA PharMetrics®️ Plus for Academics data Youths 3–18 yrs with ADHD (n = 165 794)	2007–2015	ICD-9/10 No OLU criteria	Decreased AP use: 4.4% (2007) to 3.4% (2015) Increased co-prescription stimulants-AP for females (19.5% to 28.7%) and youth 15–18 yrs (25.9% to 32.7%) Increased risk of OLU if DBD (OR^11^ = 2.5, 95% CI: 2.3, 2.7)
Egberts et al. (2022) Germany, Austria, and Switzerland	Child and adolescent psychiatric centres (n = 18) Youths 6–18 yrs with new AP use (n = 710) followed up to 6 months after treatment initiation	2014–2018	ICD-10 SPCs^12^	OLU: 80.7% of treatment episodes Risk of serious ADR^13^ not significantly different between on-label and OLU (8.7 % vs 7.5 %, p = 0.67)
Lee et al. (2022) USA	IQVIA PharMetrics®️ Plus for Academics data Youths 5–15 yrs (n = 41,098) with ADHD and no DBD at baseline	2007–2019	ICD-9/10 No indicated diagnosis	Increased risk of OLU if incident DBD (11.1%) (hazard ratio = 4.6; 95% CI 4.2–5.2) especially in younger males
Radojčić et al. (2023) UK	Clinical Practice Research Datalink (CPRD) Aurum database Youths 3–18 yrs (n = 7 217 098)	2000–2019	Clinical diagnosis No OLU criteria	Most frequent diagnoses if 1st prescription: ASD (12.7%), non-affective psychosis (8.6%), anxiety disorders (7.5%), ADHD (7.1%), depression (6.4%), conduct disorders (6.1%)
Costales et al. (2024) USA	Kaiser Permanente Northern California Outpatients youths 10–17 yrs (2021, n = 5828):	2013–2021	ICD-9/10 No FDA-approved indication (ASD, psychosis, BP, Tourette’s syndrome)	OLU in new SGA users ranged from 80.7% (2013) to 69.2% (2019) Increased OLU during COVID-19 pandemic in 10–14 yrs (post-March 2020 slope change = 0.11, p = 0.02) Most frequent diagnoses: depression, anxiety disorders, other mood disorders, ADHD
Driscoll and McCarthy (2024) Ireland	National audit of mental health services Youths < 18 yrs with AP prescription (n = 437)	2021	Clinical diagnosis ASD excluded No OLU criteria	Target conditions are more often symptoms (75.5%) than disorders: agitation (25%), irritability (18%), emotional dysregulation (14%)

1. SGA: second-generation antipsychotic; 2. ICD: international classification of diseases; 3. OLU: off-label use; 4. RCT: randomised controlled trial; 5. ADHD: attention-deficit/hyperactivity disorder; 6. AP: antipsychotic; 7. DBD: disruptive behaviour disorder; 8. ASD: autism spectrum disorder; 9. FDA: Food Drug Administration; 10. BP: bipolar disorder; 11. OR: odds ratio; 12. SPCs: Summaries of Product Characteristics; 13. ADR: adverse drug reaction.

**Table 2.** Studies exploring antipsychotic off-label use (OLU) in adults.

Authors, year Country	Population	Period	Diagnosis off-label use criteria	Main findings
Alexander et al. (2011) USA	IMS Health National Diagnostic and Therapeutic Index Outpatient visits (n = 4800 physicians)	1995–2008	Clinical diagnosis FDA^1^-approved indications in 2008	OLU^2^ all AP^3^: 74% of AP visits in 1995 (n = 4.4 million), 60% in 2008 (n = 9.0 million) OLU SGA^4^: increased from 50% in 1995 to 66% in 2003, before declining to 60% in 2008. OLU FGA^5^: declined from 78% in 1995 to 67% in 2008
Comer et al. (2011) USA	National Ambulatory Medical Care Survey Psychiatric visits for anxiety disorder (including traumatic stress disorders) (n = 4166)	1996–2007	ICD^6^-9 FDA-approved indications	OLU: 48.3% of visits with AP (n = 596, 16.7%) Increased SGA prescriptions from 3.8% (1996–1997) to 20.5% (2006–2007) (OR^7^=5.82, 95% CI = 3.4–9.8, p < 0.001) Decreased FGA prescriptions from 5.8% (1996–1997) to 1.0% (2006–2007) (OR = 0.07, 95% CI = 0.02–0.2, p < 0.001)
Leslie and Rosenheck (2012) USA	Medicaid Programs in 42 states AP users n = 372,038	2003	ICD-9 Exclusion SZ^8^, BP^9^	OLU: 57.6% Most frequent diagnoses: ‘other’ mental disorder (35.0%), minor depression (25.4%), major depression (23.2%) No diagnosis: 18.8%
Citrome et al. (2013) USA	OptumInsight commercial database Outpatients 18–64 yrs with SGA use (n = 18,352)	2008–2011	ICD-9 Exclusion SZ, BP, major depression	OLU: 19.6% Most frequent diagnoses: anxiety disorder (all SGA: around 30%), substance abuse (quetiapine 22.7%)
Marston et al. (2014) UK	Primary care database Health Improvement Network (THIN) AP use (n = 47,724)	2007–2011	Read code lists Exclusion psychotic disorder, BP	OLU: FGA from 27% to 35% by molecule, >50% total; SGA: from 38% to 64% by molecule Most frequent diagnoses: anxiety, depression, sleep disorders, dementia No diagnosis: FGA 12–17%
Driessen et al. (2016) USA	Random sample of 5% of Medicare beneficiaries SGA users (n = 76,369 in 2012)	2006–2012	ICD-9 Exclusion SZ, BP, major depression	Decline in OLU from 51% (2006) to 45% (2012) Most frequent diagnoses (2012): dementia (42%), anxiety disorders (14%), minor depression (23%) Most significant growth in prevalence from 2006 to 2012: hyperkinetic disorder (136%), anxiety disorder (113%), insomnia (102%)
Osborne et al. (2016) UK	Population-based post-marketing safety cohort survey National Health Service database Quetiapine XL users with prescription by GPs10 (n = 13,276)	2008–2013	Survey questionnaire Clinical indication no OLU criteria	OLU: 28.3% Most frequent diagnoses: psychotic disorder (11.8%) and depression (11.4%)
Gjerden et al. (2017) Norway	National prescription database Outpatients with quetiapine use (n = 47,474)	2004–2015	ICD-10 Exclusion SZ, mood disorders	OLU in users with ≥ 2 prescriptions: 60% (2015) Dose > 200 mg: 5.9% (2015) Decline (2004–2015) of quetiapine mean dose from 1.58 defined daily dose (SD 8.00) to 0.48 (SD 2.27)
Huang et al. (2019) Taiwan	National prescription database Outpatients with anxiety disorders ≥ 18 years (n = 26 235)	2005–2013	ICD-9 Exclusion SZ, BP, dementia	Increase OLU: 8.4% (2005) to 9.1% (2013) (OR= 1.21, 95%CI 1.17–1.25) Most frequent diagnoses (2013): PTSD^11^ (24%), OCD^12^ (24.4%), panic disorder (18%)
Bastaki et al. (2021) Qatar	Major healthcare providers database Qatari outpatients with AP use (n = 9,349)	2018–2020	No OLU criteria	OLU: 69% Most frequent diagnoses: depression (26%), anxiety disorders 20%
Højlund et al. (2021b) Denmark	National prescription database AP users (n = 630 307)	1997–2018	ICD-10 No OLU criteria	OLU without psychiatric, neurological or oncological disorder: 37% of prevalent and 39% of incident use OLU without SZ/BP: 66% of prevalent and 85% of incident use OLU without mood and anxiety disorders: 15% of prevalent and 26% of incident use
Horvitz-Lennon et al. (2021) USA	Medicaid and Medicare coverage in 4 states SGA users 18–64 yrs (n= 301,367)	2008–2013	ICD-10 Exclusion SZ, BP I, severe major depression	Always OLU: 37% to 42% No psychiatric diagnosis: 26% to 36%
Wang et al. (2021) China	Discharged patients (n = 981) from tertiary psychiatric hospitals (n = 41)	2019	ICD-10 Exclusion SZ, BP	OLU: 63% Most frequent diagnoses: dissociative (conversion) disorder (89.9%), organic mental disorders (81.7%), dementia (79%), OCD (78%)
Pirhonen et al. (2022) Finland	46-year survey of Northern Finland Birth Cohort 1966 (n = 7071) National prescription database	2012–2014	ICD-8/9/10 Exclusion SZ, BP	OLU: 52.4% Most frequent diagnoses: depression (70%), anxiety (40%), substance use disorder (29%) No psychiatric diagnosis: 15%
Radha Krishnan et al. (2024) Australia	Random sample (10%) of the adult Australian population GPs survey Bettering the Evaluation and Care of Health (n = 9821 AP prescriptions)	2005–2021	Clinical diagnosis No OLU criteria	OLU: 27% Most frequent diagnoses: psychological disorders other than SZ or BP (20. 6%), neurological disorders (10%)

1. FDA: Food Drug Administration; 2.AP: antipsychotic; 3. OLU: off-label use; 4.SGA: second-generation antipsychotic; 5.FGA: first-generation antipsychotic; 6. ICD: international classification of diseases; 7.OR: odds ratio; 8. SZ: schizophrenia; 9. BP: bipolar disorder; 10. GPs: General Practitioners; 11. PTSD: post-traumatic stress disorder; 12. OCD: obsessive compulsive disorder.

### Frequency and characteristics of antipsychotic OLU

#### Studies characteristics

Studies were conducted in 14 countries (Table 1): 15 were North-American (USA= 14, Canada = 1), 7 European (UK *n* = 3; Denmark *n* = 1, Germany *n* = 1, Ireland *n* = 1, Norway *n* = 1; Finland *n* = 1; Germany, Austria, and Switzerland *n* = 1), 3 Middle-East and Asian (China *n* = 1, Qatar *n* = 1, Taiwan *n* = 1), and 1 Oceanian (Australia). Four studies were carried out on data collected before 2000 (Alexander et al. 2011; Comer et al. 2011; Ronsley et al. 2013; Sohn et al. 2016).

Regarding setting and sampling method, most (*n* = 10) of the 12 studies on youths (Table 1) were performed on prescription databases, and the other two were multicentre surveys performed in mental health services (Egberts et al. 2022; Driscoll and McCarthy 2024). Of the 15 studies on adults, most (*n* = 11) were also performed on prescription databases, one in a population-based post-marketing safety cohort survey (Osborne et al. 2016), one in a tertiary psychiatric hospital (Wang et al. 2021), one in a birth cohort (Pirhonen et al. 2022) and one in a random sample of the general population (Radha Krishnan et al. 2024).

Three out of four studies (74%) concerned OLU of all antipsychotics (youths *n* = 9, adults *n* = 11) and 7 of SGA only (youths *n* = 3, adults *n* = 4). The criteria used to define OLU varied widely from one study to another (Tables 1 and 2). Approved indications/Summaries of Product Characteristics were mentioned by 7 studies (youths *n* = 5, adults *n* = 2). These criteria were applied more or less stringently from one study to another, i.e., approved indications were restricted to those listed for each antipsychotic or were broadly defined as indications approved for at least one antipsychotic. Two studies were restricted to youths with attention-deficit/hyperactivity disorder (ADHD) (dosReis et al. 2022; Lee et al. 2022) and one study excluded youths with autism spectrum disorder (Driscoll and McCarthy 2024). Eight studies performed in adults excluded varying diagnostic categories: schizophrenia and bipolar disorder were the most frequently excluded, while other studies also excluded all mood disorders or dementia (Leslie and Rosenheck 2012; Citrome et al. 2013; Marston et al. 2014; Driessen et al. 2016; Gjerden et al. 2017; Huang et al. 2019; Horvitz-Lennon et al. 2021; Wang et al. 2021; Pirhonen et al. 2022; Radojčić et al. 2023).

#### Frequency and characteristics of antipsychotic OLU in youths

Five of the 12 studies examined trends in antipsychotic OLU over periods ranging from 4 to 8 years. One reported decreasing or stable trends (dosReis et al. 2022). Three reported a slight increase in OLU (Schröder et al. 2017; Candon et al. 2021) that was restricted to the COVID-19 pandemic period in one study (Costales et al. 2024). Another study examined the incidence of antipsychotic use in youths with ADHD and no disruptive behaviour disorder (DBD) at baseline (Lee et al. 2022). The seven other studies reported data on antipsychotic OLU prevalence (Table 1).

Irrespective of the methods, all studies found a high frequency of antipsychotic OLU in youths. In most studies, antipsychotic OLU was observed in around two out of three antipsychotic users (Table 1). The highest prevalence was observed in a multinational clinical sample with antipsychotic OLU occurring in 80.7% of treatment episodes (Egberts et al. 2022).

SGA OLU predominated in youths and frequently involved drugs with labelled indications in this population (ariprazole, quetiapine, olanzapine or risperidone) (Egberts et al. 2022; Lee et al. 2022; Radojčić et al. 2023; Driscoll and McCarthy 2024).

A German study found that OLU by indication was more frequent than OLU by age (Schröder et al. 2017). The diagnoses most often concerned were ADHD, mood disorders and anxiety disorders. However, as shown by a national audit of Irish mental health services, antipsychotic OLU most often targeted symptoms rather than disorders (Driscoll and McCarthy 2024). A US study using data collected on outpatient visits found that no psychiatric diagnosis was recorded in 15% of visits with SGA prescription (Sohn et al. 2016).

#### Frequency and characteristics of antipsychotic OLU in adults

Five of the 15 studies examined trends in antipsychotic OLU over periods ranging from 6 to 13 years. Three reported decreasing trends for all antipsychotics (Driessen et al. 2016) or for first-generation antipsychotics (FGA) only (Alexander et al. 2011; Comer et al. 2011). Three reported increased OLU for all antipsychotics (Huang et al. 2019) or for SGA only (Alexander et al. 2011; Comer et al. 2011). One study reported a temporal decline in quetiapine doses (Gjerden et al. 2017). The nine other studies reported data on antipsychotic OLU prevalence (Table 2), which was most often around 30–60% but ranged from <10% in patients with anxiety disorders (Huang et al. 2019) to 75% of outpatient visits with antipsychotic prescription (Alexander et al. 2011).

FGA most often concerned by OLU were flupenthixol, haloperidol, levomepromazine, chlorpromazine, pherphenazine and sulpiride while most often concerned SGA were quetiapine, olanzapine and risperidone (Marston et al. 2014; Huang et al. 2019; Bastaki et al. 2021; Højlund et al. 2021a; Wang et al. 2021; Pirhonen et al. 2022; Radha Krishnan et al. 2024).

The psychiatric diagnoses most frequently associated with antipsychotic OLU were mood and anxiety disorders (Table 2). A substantial proportion of users (15% to 42%) had no recorded psychiatric diagnosis (Leslie and Rosenheck 2012; Marston et al. 2014; Højlund et al. 2021a; Horvitz-Lennon et al. 2021; Pirhonen et al. 2022).

### Effects of antipsychotic OLU in specific mental health conditions

#### Anxiety disorders

A systematic review and meta-analysis synthesised the studies published up to 2011 on the effects of SGA in several off-label conditions (Maher et al. 2011). Of the 14 trials assessing the efficacy of SGA in generalised anxiety disorder (GAD), 12 were placebo-controlled and five reported the percentage of responders (at least 50% reduction in Hamilton Anxiety Rating Scale score). The trials assessing the efficacy of olanzapine (*n* = 1) and risperidone (*n* = 1) did not find any significant results. The pooled results of the three quetiapine trials (dose range 50–300 mg/d) showed a 26% increase in positive response at 8 weeks (effect size 0.30). The strength of evidence was classified as moderate considering the heterogeneity (I^2^=78.2%, *p* = 0.01) and the fact that all studies were funded by the manufacturers. Of the 16 trials assessing the efficacy of SGA augmentation therapy in antidepressant-resistant obsessive-compulsive disorder (OCD), 10 were placebo-controlled and all reported the percentage of responders (25%-35% reduction on Yale-Brown Obsessive Compulsive Scale). The trials assessing the efficacy of olanzapine (*n* = 2) and quetiapine (*n* = 5) did not find any significant results. The pooled results of the three risperidone trials (dose range 0.50–2.25 mg/d) showed a 4-fold greater likelihood of response with risperidone (effect size 1.14). The strength of evidence was classified as moderate because of a potential publication bias.

More recently, an umbrella review synthesised 25 systematic reviews (of which 4 included a meta-analysis) up to 2020 investigating the efficacy of antipsychotics in several anxiety disorders (Garakani et al. 2024): GAD (*n* = 13), panic disorder (*n* = 8), social anxiety disorder (*n* = 4), phobias (*n* = 6), OCD (*n* = 3), and post-traumatic stress disorder (PTSD) (*n* = 2). Only one review was found to be of high quality, the others were rated as being of low (*n* = 4) or critically low (*n* = 20) quality. Efficacy of quetiapine monotherapy in GAD was the only finding with demonstrated evidence. There was no convincing evidence regarding the efficacy of antipsychotics in other disorders.

A study performed on the Taiwan national prescription database (Huang et al. 2019) (Table 2) provided information on real-life antipsychotic prescribing practices in anxiety disorders. A significant increase in antipsychotic OLU rates was observed in adults with anxiety disorders (8.4% in 2005 to 9.1% in 2013, OR= 1.21, 95%CI 1.17–1.25). The increase was more marked in patients with comorbid depression (62%) than in those without depression (20%). The frequency of antipsychotic OLU was higher in patients treated by ­psychiatrists (15.3%) than in those treated by non-psychiatrists (6.8%). Although increased use concerned all anxiety disorders, the highest prevalence was found for PTSD (24%), OCD (24.4%) and panic disorder (18%). Quetiapine was the most frequently prescribed SGA for patients with anxiety, with increasing rates from 0.3% in 2005–2017 to up to 0.9% in 2011–2013 (OR = 2.98, 95%CI: 2.60–3.42).

#### Personality disorders

A systematic review including 57 articles published up to 2011 examined the evidence on SGA efficacy in personality disorders (Rosenbluth and Sinyor 2012). Most studies were open trials with very small samples of persons with borderline personality disorder (BPD). The authors concluded that there was no evidence regarding the efficacy of SGA OLU on overall illness severity of personality disorders and recommended restricting prescription to persons presenting a comorbid condition with a labelled indication of SGA. However, they highlighted the potential interest of clozapine for reducing self-harm/suicidal behaviour in BPD.

A recent Cochrane review synthesised the findings of RCTs published up to 2022 on the efficacy of FGA (*n* = 6) or SGA (*n* = 16) in BPD (Stoffers-Winterling et al. 2022). Reduction of anger was significantly greater with antipsychotics vs. placebo (standardised mean difference (SMD) −0.37, 95% CI −0.55 to −0.18; *p* = 0.0001). No evidence was found regarding the effects of antipsychotics vs. placebo on BPD symptom severity (SMD -0.18, 95% CI -0.45 to 0.08; *p* = 0.18); self-harm (Relative risk (RR) 0.66, 95% CI 0.15 to 2.84; *p* = 0.57); suicide-related outcomes (SMD 0.05, 95% CI –0.18 to 0.29; *p* = 0.67); psychosocial functioning (SMD 0.05, 95% CI -0.18 to 0.29; *p* = 0.67); impulsivity (mean difference (MD) 0.46, 95% CI −12.93 to 13.85; *p* = 0.95).

The STAR Network Depot study was carried out on a naturalistic cohort of 449 patients initiating long-acting injectable (LAI) antipsychotic treatment (D’Agostino et al. 2022). Patients were recruited over one year in 35 territorial and university mental health departments in Italy. One out of four patients (26%) was prescribed LAI OLU, of whom 23.7% had a diagnosis of personality disorder.

#### Sleep disorders

A systematic review and meta-analysis synthesised the findings up to 2022 of RCTs (*n* = 19) and cross-over trials (*n* = 2) assessing the effects of quetiapine ≤ 300 mg on sleep quality (Lin et al. 2023). Quetiapine monotherapy was assessed in most trials (*n* = 19). Studies were performed in persons with depression (*n* = 9), anxiety disorders (*n* = 5), other diseases (*n* = 5) or healthy persons (*n* = 3). All used a standardised measure of sleep quality (most often the Pittsburgh Sleep Quality Index *n* = 15). Fewer than half of the trials had a low risk of bias, and three out of four trials were funded by pharmaceutical companies. Sleep quality was significantly improved in the quetiapine group vs. placebo for GAD (SMD -0.59, 95% CI –0.92 to –0.27, high heterogeneity I^2^ = 92%, *p* *<* 0.00001) and depression (SMD –0.47, 95%CI: –0.66 to –0.28, high heterogeneity I^2^ = 71%, *p* *<* 0.007) as well as in healthy persons (SMD –1.33, 95%CI –2.12 to –0.54). Improvement in sleep quality was observed at doses 50 mg/day (SMD –0.36, 95%CI –0.60 to –0.11, 150 mg/day (SMD –0.4, 95%CI –0.52 to –0.29), and 300 mg/day (SMD –0.17, 95%CI: –0.31 to –0.04), but not at 25 mg/day (SMD –0.38, 95%CI –1.11 to 0.34)

### ADR associated with antipsychotic OLU

The increased risk of ADR associated with antipsychotic OLU is emphasised by almost all studies. A meta-analysis synthesised the findings of RCTs (*n* = 35) published up to 2019 providing a comparison of metabolic outcomes with SGA OLU vs. placebo (Stogios et al. 2022). The RCTs included adults (18–65 years) with psychiatric diagnoses without a labelled indication for antipsychotics. Across all diagnoses, significantly greater weight gain was found for olanzapine (MD = 3.24 kg, 95% CI 2.57 to 3.90, *p* < 0.00001, I^2^=63%). Trends in the same direction were identified for risperidone (MD = 0.66 kg, 95% CI −0.09 to 1.40, *p* = 0.08, I^2^ =  57%) and quetiapine (MD = 0.82 kg, 95% CI −0.02 to 1.65, *p* = 0.06, I^2^=96%). The effects of other SGA on weight could not be determined because of the small number of studies.

ADR induced by antipsychotic OLU in children and adolescents are of great concern, especially regarding metabolic ADR which can lead to dramatic increase in body mass index (Hoekstra and Dietrich 2022). A multinational study included youths (6–18 years, *n* = 710) followed up 6 months after antipsychotic initiation (Egberts et al. 2022). The frequency of serious ADR rated with the Paediatric Adverse Event Rating Scale was not significantly different between on-label and OLU antipsychotics (8.7% vs 7.5%, *p* = 0.67).

Several studies specifically addressed ADR related to quetiapine OLU. Meta-analyses and systematic reviews reported a higher risk of weight gain, hypertriglyceridaemia, high-fasting glucose increase, sedation and hyperprolactinaemia with quetiapine vs. placebo (Garakani et al. 2022; Lin et al. 2023). A population-based study performed on Danish health registers explored the risk of diabetes associated with low-dose quetiapine (25–50 mg) (Højlund et al. 2021b). New quetiapine users ≥18 years (*n* = 54 616) without severe mental illness (SMI) were matched with new users of selective serotonin reuptake inhibitors. The cumulative incidence of type 2 diabetes (first diagnosis/antidiabetic medication/haemoglobin A1C (HbA1C) ≥6.4%) did not differ between the two groups (incidence rate ratios (IRR) for as-treated 0.99; 95%CI 0.87 to 1.13; for intention to treat 0.92, 95%CI 0.84 to 1.00). Sensitivity analyses showed that higher cumulative doses were associated with an increased risk of diabetes when persons using higher tablet strengths (>50 mg as proxy for higher daily doses) were included (OR 1.44, 95%CI, 1.13–1.84).

Another study on the same population examined the impact of initiating low-dose quetiapine on HbA1c, triglyceride and cholesterol levels (Højlund et al. 2023). Metabolic monitoring before and after initiation was performed in a very small proportion of newly eligible quetiapine users (*n* = 31 208) ranging from 1.2% for fasting triglycerides to 9.3% for total cholesterol. No significant increase in HbA1c level was found after quetiapine initiation in the total sample (β = 0.999, 95%CI 0.997 to 1.002), but an increase was observed in patients with normal level at baseline vs. those with prediabetic or diabetic HbA1c levels before initiation (β = 1.006 vs. β = 0.999/0.971; *p* < 0.001). Initiation of low-dose quetiapine was associated with decreased levels of total cholesterol (β = 0.993, 95%CI 0.98 to 0.996), low-density lipoprotein cholesterol (β = 0.984, 95%CI 0.977 to 0.990), high-density lipoprotein cholesterol (β = 0.982, 95%CI 0.98 to 0.99) and with increased levels of fasting triglycerides (β = 1.04, 95%CI 1.027 to 1.072).

A Swedish population-based study was carried out in adults ≥ 18 years with ≥2 prescriptions of low-dose olanzapine (≤ 5 mg/d) and/or quetiapine (≤ 75 mg/d) (Berge et al. 2022). Persons with SMI or a prescription of antipsychotics or cardiometabolic drugs were excluded. The risk of cardiometabolic mortality was similar in persons exposed to low doses of olanzapine or quetiapine vs. those unexposed to antipsychotics (hazard ratio (HR) 0.86, 95% CI 0.64 to 1.15). However, the risk varied with duration of exposure: it was significantly lower for those treated for *<*6 months (HR 0.56, 95% CI 0.37 to 0.87) and significantly higher for those treated for 6–12 months (HR 1.89, 95% CI 1.22 to 2.92), suggesting that a selection bias may lead to more antipsychotic prescription to persons with lower cardiometabolic risk profiles.

An audit of 270 New Zealand general practitioners in 2015–2016 found that metabolic monitoring was seldom performed in quetiapine users: only 2.3% of them had full monitoring, while 68% had partial monitoring and 29.7% no monitoring at all (Huthwaite et al. 2018).

### Studies on prescribers

A non-industry funded survey mailed to 300 Italian psychiatrists (67% response rate) explored SGA OLU (Aguglia et al. 2021). Although most reported SGA OLU (very often: 16.7%, often: 33.4% or occasionally: 34.7%), only half of them routinely required users’ informed consent before SGA initiation. The main motives for SGA OLU were literature evidence (51.5%), non-response (37.2%) or ADR (11.9%) with labelled drugs, or indications from other regulatory agencies such as the Food and Drug Administration (FDA). Patient preference was mentioned only by 1.5% of participants.

A study performed on a 20% random sample of the Danish population investigated quetiapine prescribing trends from 2001–2020 (Højlund et al. 2022). Although the study did not provide a quantitative estimate of quetiapine OLU, several indicators suggested that these prescribing practices increased in GPs’ prescriptions over the study period. In 2020, half (53%) of quetiapine prescriptions were initiated by GPs, especially in elderly patients (20% in those ≤17 years vs. 72% in those aged ≥80 years). The mean dose of quetiapine was 43 mg/d in users aged ≥ 80 years.

A qualitative study on 15 Canadian GPs found they did not consider quetiapine as a first-line treatment and restricted its use to patients unresponsive to other drugs and/or presenting with complex psychosocial needs in order to ‘keep patient functioning’ (Kelly et al. 2018). Quetiapine was preferred to benzodiazepines because it was considered as non-addictive. Most participants considered low-dose quetiapine as safe and were not aware of mechanisms of action or of guidelines especially regarding monitoring. Quetiapine use was influenced by the prescribing practices of other colleagues, but prescribers did not consider that promotion from pharmaceutical companies impacted their prescribing practices.

A survey including 16 prescribers investigated the reasons for not discontinuing long-term antipsychotic OLU in persons with neurodevelopmental disorders (de Kuijper and Hoekstra 2017). Of the 3,299 persons living in residential facilities in the Netherlands, 29.6% used antipsychotics, most often (95%) prescribed for more than one year for off-label indications. According to the prescribers, half (51%) of these long-term prescriptions could be discontinued. A higher likelihood of discontinuation was associated with prescription for challenging behaviour. Non-discontinuation was most often based on the prescriber’s decision but lack of consent of the legal representative was also frequent (up to 30% in some facilities). Differences in attitudes and beliefs of settings’ staff were identified by the authors as impacting prescribing patterns and decisions of physicians not to discontinue OLU use.

### Impact of marketing and regulatory issues

Several pharmaceutical companies have been convicted in the USA for marketing antipsychotic OLU (Tanne 2010, 2012; Wang et al. 2016; Wang et al. 2017). The companies promoted antipsychotic OLU in a range of unapproved uses, such as dementia in elderly people, schizophrenia and bipolar disorder in youths, depression, anxiety disorders, and insomnia. The promotion strategies included influencing the narrative of company speakers, sponsoring medical educational programs, conducting studies on unapproved uses, ghost-writing articles, providing child psychiatrists and general practitioners with free samples of the drug, and rewarding prescribers with lucrative consulting agreements (Tanne 2010, 2012; Roehr 2012).

The impact of findings obtained by small exploratory studies on OLU rates has been addressed by a systematic meta-epidemiological review (Grabitz et al. 2024). Half of the 176 studies reporting efficacy outcomes for quetiapine in unapproved indications over the period 1995–2022 were industry-funded. A positive efficacy endpoint was found by 87% of the 147 exploratory studies and 72% of the 29 confirmatory studies. According to the authors, ‘clinical agnosticism’ encouraging OLU is induced by small exploratory studies: they suggest that a drug might be effective in unapproved indications even if evidence of efficacy from controlled studies is lacking. Indeed, the authors identified nine clinical practice guidelines recommending the use of quetiapine in indications only documented by exploratory studies, such as PTSD and eating disorders.

The characteristics of antipsychotic OLU in a given country are directly related to the decisions of national regulatory agencies, since approved indications for a given drug vary widely from one country to another (Zhu et al. 2018). However, the agencies’ decisions after first market authorisation have a more limited impact on antipsychotic OLU. A study carried out on the Optum Research Database assessed trends in antipsychotic OLU rates after FDA approval in 2009 of supplemental indications in youths for olanzapine and quetiapine and non-approval of expanding indications for ziprasidone (Wang et al. 2016). After the FDA decisions, no significant differences were found in the rates of new prescriptions in youths compared to adults favouring drugs with new indications and cautioning against ziprasidone. Another study performed on the same database found no reduction in olanzapine OLU after settlements for illegal promotion of this drug in OLU indications (Wang et al. 2017).

Conversely, a slight reduction in antipsychotic OLU in youths was observed in the year following the implementation of a state Medicaid peer-review authorisation programme (5–9 years: 88.4 to 85.7%; 10 to 17 years: 74.2% to 71.8) (Pennap et al. 2018). This programme was based upon a review of new antipsychotic prescriptions by clinical pharmacists on the basis of clinical criteria such as age, diagnosis, target symptom severity, medication dosage, monitoring criteria, etc.

## Discussion

The recent literature on antipsychotic OLU confirms the persisting high prevalence of these prescribing practices. Increased rates of antipsychotic OLU became a source of concern soon after the introduction of SGA, especially in youths (Zito et al. 2000; Verdoux et al. 2010; Patten et al. 2012; Olfson et al. 2015; Hálfdánarson et al. 2017). Three decades later, antipsychotic OLU rates are still very high, since two out of three youths and 30–60% of adults using antipsychotics have no labelled indications. However, there may be a ceiling effect as some studies are reporting stable or decreasing trends of antipsychotic OLU rates (Driessen et al. 2016; dosReis et al. 2022).

The reasons for OLU in the recent literature are comparable to those reported by older studies (Olfson et al. 2015; Verdoux et al. 2015). Regarding OLU by indication or age, changes are mostly related to decisions made by regulatory agencies that authorise supplemental indications rather than to modifications of prescribing practices. The most frequent diagnoses are still ADHD, mood and anxiety disorders in youths, and mood and anxiety disorders in adults. In both populations, a substantial proportion of antipsychotic users (15–40%) have no recorded psychiatric diagnosis (Leslie and Rosenheck 2012; Marston et al. 2014; Sohn et al. 2016; Højlund et al. 2021a; Horvitz-Lennon et al. 2021; Pirhonen et al. 2022).

The most striking change in recent years concerns the growing body of literature on quetiapine OLU (Tanne 2010; Osborne et al. 2016; Gjerden et al. 2017; Huthwaite et al. 2018; Kelly et al. 2018; Huang et al. 2019; Højlund et al. 2021b; Berge et al. 2022; Højlund et al. 2022; Højlund et al. 2023; Lin et al. 2023; Garakani et al. 2024; Grabitz et al. 2024). A consistent and widespread finding is that rates of low-dose quetiapine OLU are increasing for anxiety or sleep symptoms in persons without a SMI or psychiatric disorder.

Quetiapine prescribing trends may be viewed as paradigmatic to illustrate the multifactorial determinants of antipsychotic OLU. These prescribing practices are undoubtedly facilitated by the illegal promotion of OLU by pharmaceutical companies through sponsored medical educational programs, exploratory studies on unapproved uses, the ghost-writing of articles, etc. (Tanne 2010; Wang et al. 2016; Grabitz et al. 2024). Guidelines recommending the use of quetiapine in indications documented only by exploratory studies also contribute to OLU (Garakani et al. 2024; Grabitz et al. 2024). Attitudes of prescribers leading to a feeling of innocuousness and extension of perceived indications also favour OLU (Verdoux et al. 2010; Huthwaite et al. 2018; Kelly et al. 2018; Højlund et al. 2022). Prescribing low-dose quetiapine may be viewed as a common practice for anxiety or sleep symptoms. Prescribers may consider that quetiapine is less risky than drugs with labelled indications such as benzodiazepines; because they underestimate the risk of adverse events and are not aware of the need for metabolic monitoring (Marston et al. 2014; Hoekstra and Dietrich 2022; Højlund et al. 2022; Stogios et al. 2022; Højlund et al. 2023). Furthermore, prescribers are often unaware that they may be responsible for the legal consequences of ADR induced by antipsychotic OLU (Martin-Latry et al. 2007).

As illustrated by the case of quetiapine, there is little doubt regarding the need to reduce antipsychotic OLU as a first-line strategy in persons without SMI because of its detrimental benefit-risk ratio. However, antipsychotic OLU may be necessary in some psychiatric conditions (Wang et al. 2021; Driscoll and McCarthy 2024). These situations have characteristics mirroring those described for the case of quetiapine. They mostly concern persons with resistant psychiatric disorders or presenting with serious ADR with labelled drugs. The potential clinical benefits clearly predominate over the financial benefits for the drug company. The prevalence of antipsychotic OLU for resistant disorders is not documented.

Antipsychotic OLU may be regarded as a spectrum of prescribing practices, with low-dose quetiapine as the first-line treatment of sleep symptoms at the one end, and at the other, clozapine as a fifth- or even tenth-line treatment in refractory and severe personality (Rosenbluth and Sinyor 2012) or neurodevelopmental disorders (Verdoux et al. 2024). A common point between these two opposite situations is that evidence-based studies may also be lacking for OLU prescriptions motivated by resistance. However, in such cases it should be borne in mind that ‘there will always be individuals that achieve significant improvement from a therapy despite limited controlled evidence’ (Faden and Citrome 2019).

## Conclusion

Although OLU is common in most medical specialties, it is highly prevalent in psychiatry owing to the lack of clear diagnostic boundaries and limited pathophysiological knowledge (Martin-Latry et al. 2007; Verdoux 2018; Wang et al. 2021). Addressing the influence of the pharmaceutical industry on the OLU prescription of newly marketed drugs is a clear public health priority. OLU may also be reduced by making prescribers more aware of industry marketing practices, especially regarding the limitations of industry-sponsored exploratory studies (Wang et al. 2017; Grabitz et al. 2024).

However, antipsychotic OLU should be neither trivialised nor demonised, as it remains the only therapeutic option in several clinical conditions. Due to the high cost and complexity of conducting RCTs, not all potential indications can be tested, especially in resistant psychiatric disorders or youth/adolescent populations, where RCTs are particularly challenging.

The literature is scarce regarding patients’ opinions on antipsychotic OLU. Users need to be informed not only about the benefits but also about the risks associated with OLU (Huthwaite et al. 2018; Kelly et al. 2018). Close relatives and legal representatives of the most vulnerable patients also need to be aware of when and why antipsychotics should be initiated or discontinued in non-labelled indications (de Kuijper and Hoekstra 2017). Further studies are required regarding the stigma potentially associated with antipsychotic use in non-psychotic conditions, especially in youths (King and Voruganti 2002).

## Supplementary Material

DICN24_AP_OLU_suppl_Figure (clean).docx
