# Supplementary material for: Antipsychotic off-label use in the 21st century: An enduring public health concern
Source: Dialogues Clin Neurosci. 2025 Jan 10;27(1):1–12. doi: 10.1080/19585969.2025.2449833 (PMC11789223; doi:10.1080/19585969.2025.2449833)
Supplement: DICN24_AP_OLU_suppl_Figure (clean).docx [file TDCN_A_2449833_SM8793.docx]

**Supplementary Figure 1. Study selection**

Articles identified through Medline (n=812) and PsycINFO (n=263) searching

Articles after duplicates removed

(n = 667)

Title/abstract screened

(n = 667)

Full-text articles assessed for eligibility

(n = 96)

Articles included in qualitative synthesis

(n = 51)

Articles excluded

(n = 571)

Full-text articles excluded

(n = 45)

Additional full text articles identified by hand search

(n = 2)
